# Placenta‐Derived Mesenchymal Stromal‐Like Cells Promote 3D‐Engineered Muscle Tissue Differentiation and Vessel Network Maturation

**DOI:** 10.1002/smsc.202400228

**Published:** 2024-08-06

**Authors:** Anna Tsukerman, Majd Machour, Margarita Shuhmaher, Eliana O. Fischer, Hagit Shoyhet, Orit Bar‐Am, Gali Guterman Ram, Lior Debbi, Dina Safina, Shulamit Levenberg

**Affiliations:** ^1^ Faculty of Biomedical Engineering Technion‐Israel Institute of Technology Haifa 3200003 Israel; ^2^ Interdisciplinary Program for Biotechnology Technion‐Israel Institute of Technology Haifa 3200003 Israel; ^3^ Multidisciplinary Program for Nanoscience and Nanotechnology Technion‐Israel Institute of Technology Haifa 3200003 Israel

**Keywords:** muscle implantation, placenta‐derived mesenchymal stromal cells, regenerative medicine, skeletal muscle, tissue engineering, vascularization

## Abstract

Placental‐derived stromal‐like cells (PLX‐PAD) have been shown to facilitate muscle tissue recovery after injury and stimulate angiogenesis. This work assesses the impact of PLX‐PAD cells on the vascularization and maturation of engineered skeletal muscle tissue. Specifically, their effects in direct co‐culture with endothelial cells, pericytes, and myoblasts seeded within microporous 3D scaffolds are characterized. Additionally, the impact of hypoxic PLX‐PAD cell‐conditioned medium (CM) on vascularization and muscle differentiation of engineered tissue is monitored. Co‐culture of PLX‐PAD with myocytes stimulated myocyte differentiation while PLX‐PAD CM promoted the formation of vascular networks. Implantation of a multi‐culture system of vascularized human skeletal muscle tissue and PLX‐PAD into a rectus abdominal defect in nude mice promoted myocyte differentiation, host vessel penetration, and tissue integration. These findings indicate the ability of placenta‐derived cells to induce the formation of vascularized engineered muscle constructs with potential therapeutic applications.

## Graphical abstract

Research demonstrates that placenta‐derived stromal‐like cells (PLX‐PAD) enhance vascularization and maturation in engineered skeletal muscle tissues. This study reveals that PLX‐PAD cells promote muscle differentiation and vascular network formation in co‐culture with myoblasts, endothelial cells, and support cells in 3D scaffolds. Such advancements suggest potential therapeutic applications in muscle tissue regeneration and recovery.

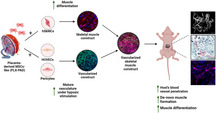

## 1 Introduction

Placenta‐derived mesenchymal stromal‐like (PLX‐PAD) cells, derived from human donor placentas express typical mesenchymal stromal cell markers,^[^ ^1^ ^]^ however, they have minimal ability in‐vitro to differentiate into cells of a mesodermal lineage.^[^ ^2^ ^]^ Recent studies demonstrated that these cells secrete diverse factors that induce angiogenesis, have immunomodulatory activities, and promote muscle tissue regeneration.^[^ ^2^, ^3^, ^4^, ^5^ ^]^ Specifically, research that focused on muscle regeneration, indicated they have positive effects as a cell therapy for mild muscle injuries upon cell injection into critical limb ischemia models.^[^ ^2^, ^3^ ^]^ However, severe muscle injuries that result in volumetric muscle loss require tissue transplantation and cannot be restored through cell injection alone.^[^ ^6^, ^7^ ^]^

The current gold standard treatment for volumetric muscle loss involves the use of autologous muscle flaps, prepared from healthy muscle tissue harvested from the patient and transplanted to the injured site. This method eliminates the risk of immune rejection but has drawbacks, including donor site morbidity and limited muscle availability.^[^ ^8^ ^]^

Emerging tissue‐engineering techniques enable the generation of functional tissue constructs that closely resemble native tissue in vitro and are capable of replacing and regenerating damaged tissues and organs.^[^ ^6^, ^7^, ^9^ ^]^ Previous studies have shown successful fabrication of transplantable muscle‐engineered tissue, prepared by seeding myoblasts onto 3D biodegradable scaffolds and differentiating them into myofibers.^[^ ^7^, ^10^, ^11^ ^]^ Yet, after transplantation, there is a critical time frame during which the host vasculature must supply the tissue with blood, oxygen, and nutrients while removing waste products to prevent ischemia, injury, and necrosis.^[^ ^12^ ^]^ The host vasculature alone is incapable of providing the required nutrient supply and gas exchange to the engineered implant. Establishing a functional engineered vascular network before implantation can enhance cell viability during tissue growth, promote structural organization, facilitate integration upon implantation, and increase overall chances of survival and of graft integration.^[^ ^1^, ^11^, ^13^, ^14^ ^]^ This can be done by co‐culturing ECs together with supporting cells (SCs), such as pericytes, fibroblasts, or mesenchymal stromal cells (MSCs).^[^ ^15^, ^16^, ^17^, ^18^, ^19^, ^20^, ^21^ ^]^ Another challenge in muscle tissue engineering presents rapid and efficient myoblast differentiation. While establishing a vascular network typically requires around a week, myoblast differentiation takes at least double the time.^[^ ^18^, ^22^, ^23^, ^24^, ^25^ ^]^ Reducing the time and increasing the efficiency of the engineered tissue maturation is critical to benefit the patients and reduce the cost of the fabrication process. Therefore, an important milestone toward achieving muscle tissue engineering is establishing conditions for a stable and functional vascular network while simultaneously promoting rapid and efficient muscle tissue differentiation.

As PLX‐PAD cells were shown to process both angiogenic activity and promote muscle differentiation, and also originate from an easily accessible cell source, we hypothesized that adding PLX‐PAD cells in the process of vascularized skeletal muscle tissue engineering will enhance in vitro maturation and vascularization, as well as the post‐transplantation integration of the 3D vascularized muscle implants. The current work aimed to examine the impact of PLX‐PAD cells on vascular network maturation and the differentiation of skeletal muscle within 3D microporous constructs.

## 2 Results

### 2.1 PLX‐PAD Cells Stimulate Human Skeletal Muscle Cell Differentiation in a 3D Construct

**Figure 1:**
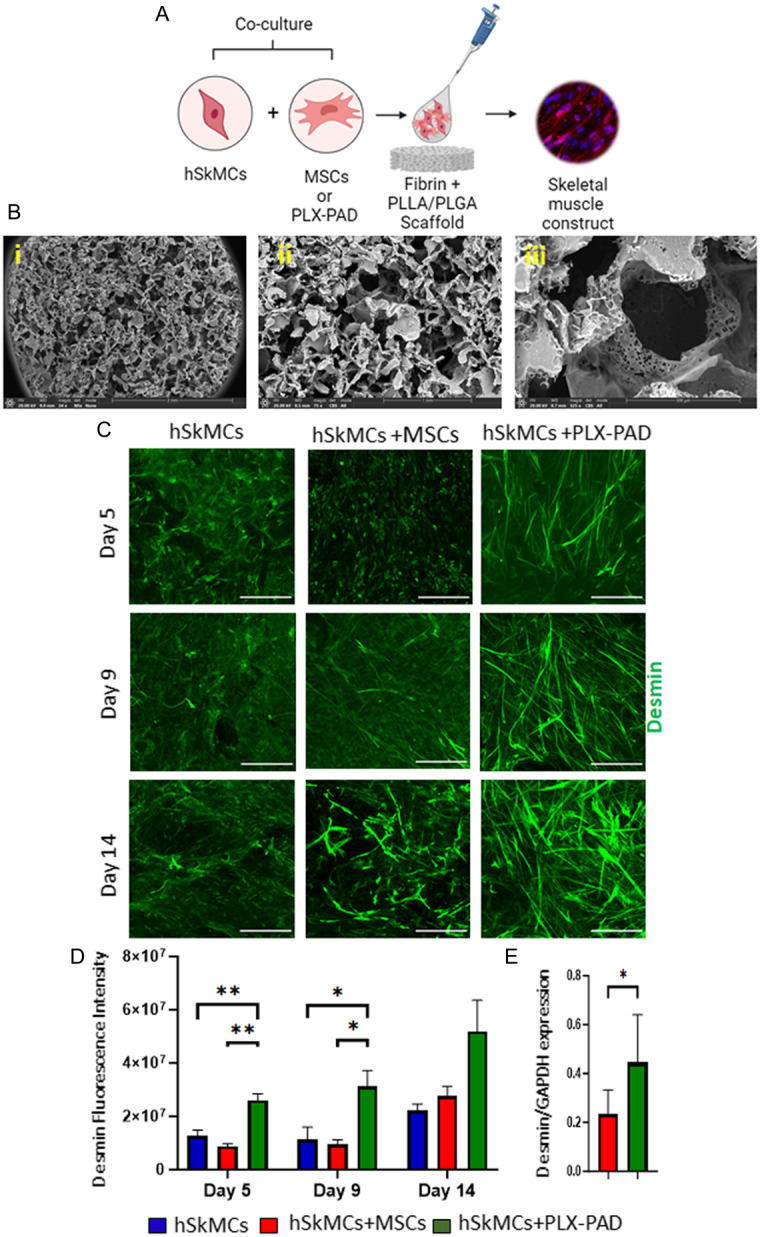
PLX‐PAD cells stimulate hSkMC differentiation. A) Schematic presentation of the experimental procedure. hSkMCs were co‐seeded with PLX‐PAD cells or MSCs on PLLA/PLGA scaffolds. B) Scanning electron microscopy (SEM) images of a PLLA/PLGA scaffold: i) ×34 magnification, ii) ×75 magnification and iii) ×325 magnification. C) Representative confocal microscopy images of PLLA/PLGA scaffolds embedded with hSkMC monoculture, hSkMC and PLX‐PAD cell co‐culture or hSkMCs and MSCs co‐culture, and stained for desmin on days 5, 9, and 14 post‐seeding. Scale bar = 200 μm. D) Desmin fluorescence intensity. Significance of differences was assessed by a two‐way ANOVA test. Data are presented as mean ± SD *n* ≥ 4. *p < 0.05, **p < 0.01, ****p < 0.0001. E) Desmin protein expression level in hSkMCs in co‐culture with PLX‐PAD cells or with MSCs. Significance of differences was assessed by *t*‐test, *n* = 7–9. *p < 0.05, **p < 0.01.

Skeletal muscle differentiation is a highly controlled and multistep process. To evaluate the ability and efficiency of PLX‐PAD to stimulate skeletal muscle cell differentiation, human skeletal muscle cells (hSkMCs) were co‐seeded within a fibrin hydrogel with PLX‐PAD cells or with adipose‐derived MSCs (referred here as MSCs) as a control, on highly microporous, 3D biodegradable PLLA/PLGA scaffolds (**Figure** **1**A). The PLLA/PLGA material has been approved for clinical applications and is well‐studied.^[^ ^26^ ^]^ These scaffolds provide mechanical strength and tunable stiffness by changing the PLLA versus PLGA ratios within the scaffolds, which we have previously reported as influential in regulating cell organization and differentiation.^[^ ^27^ ^]^ The PLLA/PLGA scaffolds have multiple levels of high porosity and interconnectivity with larger pores in the range of 212–600 μm^[^ ^28^ ^]^ (Figure 1B). Fluorescence intensity quantification showed significantly higher desmin expression levels in the PLX‐PAD‐hSkMCs as compared to the hSkMCs monoculture and MSCs‐hSkMCs scaffolds (Figure 1C,D and S1A,B, Supporting Information). Similarly, protein expression quantification showed PLX‐PAD‐hSkMCs constructs had substantially higher desmin expression levels (Figure 1E and S1C, Supporting Information), suggesting that PLX‐PAD cells significantly promote the differentiation of hSkMCs within a 3D scaffold.

### 2.2 PLX‐PAD Cells Provide Less Support for HUVECs Microvasculature Formation in 3D Construct Compared to MSCs and Pericytes

**Figure 2:**
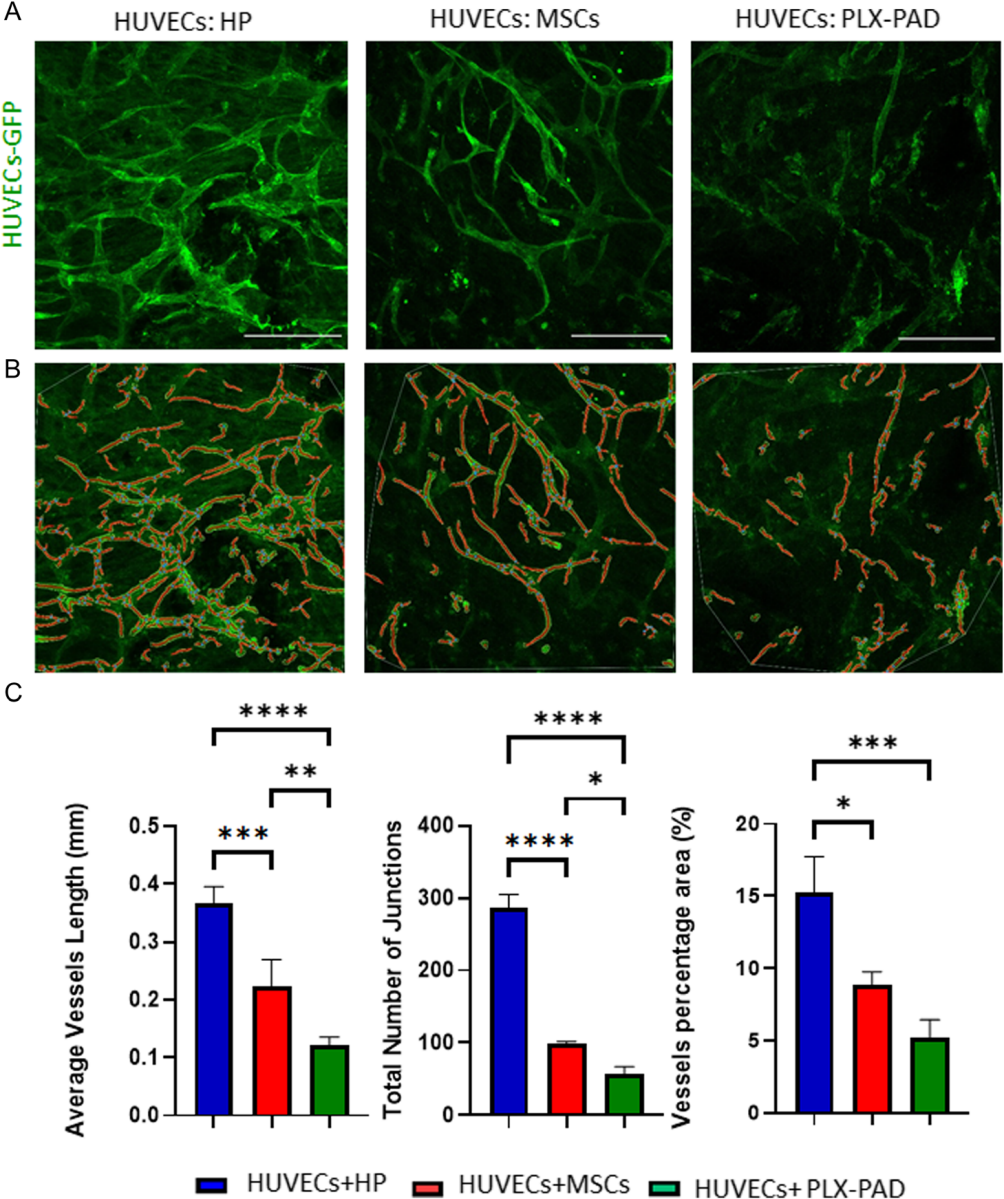
Vascular network generation by a co‐culture of HUVECs and PLX‐PAD. A) Representative confocal images of PLLA/PLGA scaffolds embedded with HUVECs‐GFP and different supporting cells, on day 7 post‐seeding. Scale bar = 200 μm. B,C) Average vessel length, total number of junctions, and vessel percentage area were calculated using the AngioTool program. Data are represented as mean ± SD, *n* ≥ 4 per group. Significance of the differences across the conditions was estimated by two‐way ANOVA. *p < 0.05, **p < 0.01, ***p < 0.001, ****p < 0.0001.

A co‐culture system of EC and mural‐supporting cells can promote the formation of a stable vascular network. To assess the capacity of PLX‐PAD cells to serve as a supportive cell, the cells were co‐seeded within fibrin hydrogel on PLLA/PLGA scaffolds, and vasculature formation was monitored. On day 7 post‐seeding, the scaffolds bearing HUVECs and human pericytes (HP) exhibited a more developed vessel network than scaffolds with HUVECs and MSCs. Those carrying PLX‐PAD as support cells showed no vasculature formation, and the HUVECs remained as single cells (Figure S2A, Supporting Information). To improve the vascular network formation, an ECM hydrogel mix was formulated and embedded with cells. HUVECs co‐cultured with HP on a PLLA/PLGA scaffold within an ECM hydrogel mix, formed a more developed vascular network as compared to scaffolds with fibrin or collagen (Figure S2B–D, Supporting Information). The vascular network proved more developed and mature than those formed in scaffolds co‐seeded with HUVECs and MSCs or PLX‐PAD (**Figure** **2**A–C).

### 2.3 PLX‐PAD Cells Stimulate Human Skeletal Muscle Cell Differentiation in a Multi‐culture System of Vascular Skeletal Muscle

**Figure 3:**
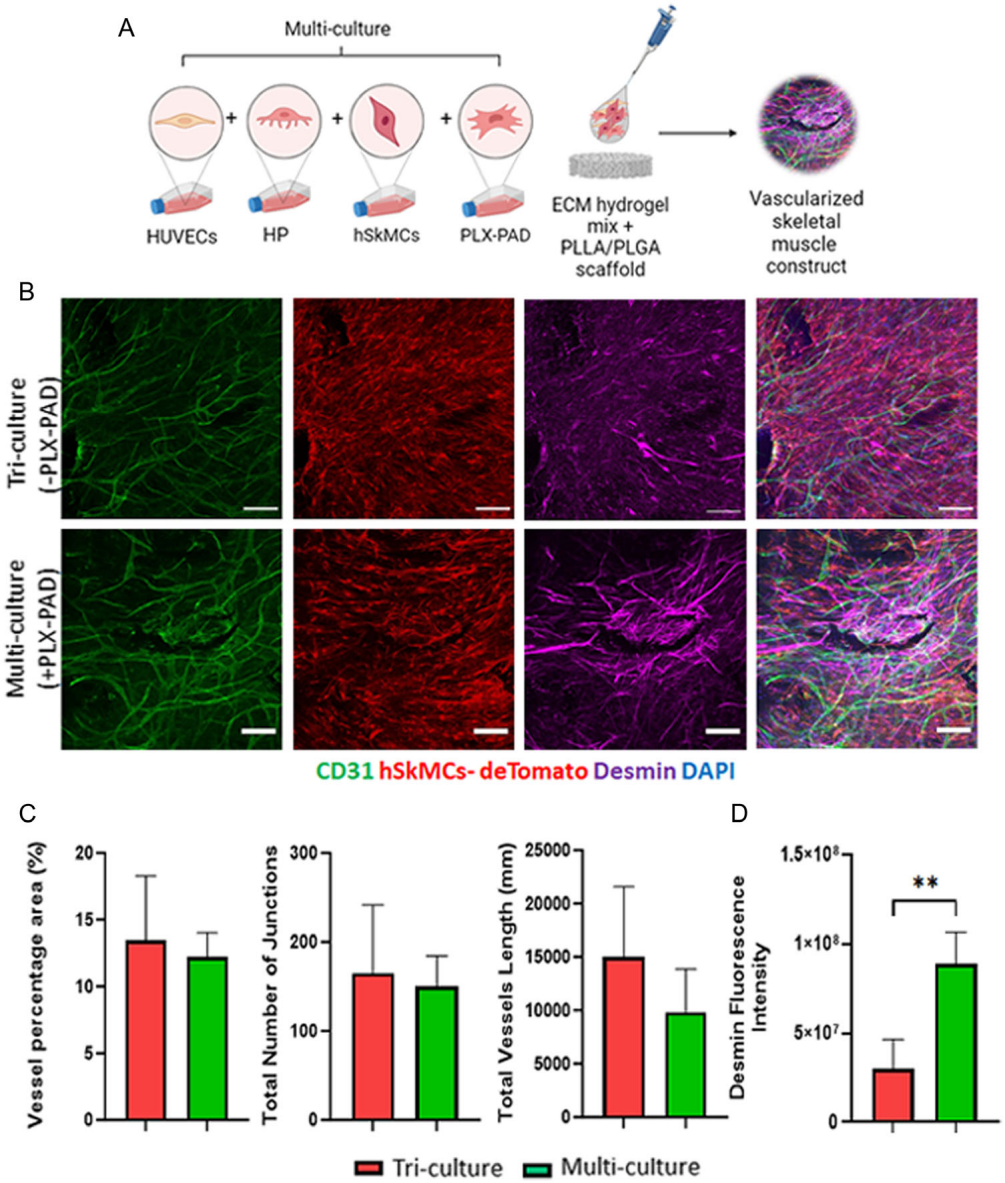
PLX‐PAD cells stimulate human skeletal muscle cell differentiation in a multi‐culture system. A) Schematic presentation of the experimental procedure. hSkMCs were co‐seeded with endothelial cells, supporting cells, with or without PLX‐PAD cells, and hydrogel ECM mix on PLLA/PLGA scaffolds. B) Representative confocal microscopy images of PLLA/PLGA scaffolds embedded with HUVECs, HP, and hSKMCs, with and without PLX‐PAD cells and stained for CD31 (green), hSKMCs‐tdTomato (red) and desmin (magenta) on day 10 post‐seeding. Scale bar = 200 μm. C) Average vessel length, total number of junctions and vessel percentage area were calculated using the AngioTool program. D) Desmin fluorescence intensity quantification. Data are presented as mean ± SD, *n* ≥ 4 per group. Statistical significance of differences across the conditions was assessed by *t*‐test. **p < 0. 01.

To examine the effect of PLX‐PAD cells in engineering composite construct of vascularized skeletal muscle tissue, hSkMCs expressing tdTomato were cultured with HUVECs and HP, with or without PLX‐PAD cells on PLLA/PLGA scaffolds (**Figure** **3**A). After 10 days both a tri‐culture system (without PLX‐PAD) and a multi‐culture system (with PLX‐PAD) contained similar vascular networks (Figure 3B,C), with significantly higher desmin expression measured in the presence of the PLX‐PAD cells (Figure 3B,D).

### 2.4 Conditioned Medium of PLX‐PAD Grown under Hypoxia Stimulates Vascular Network Maturation

**Figure 5:**
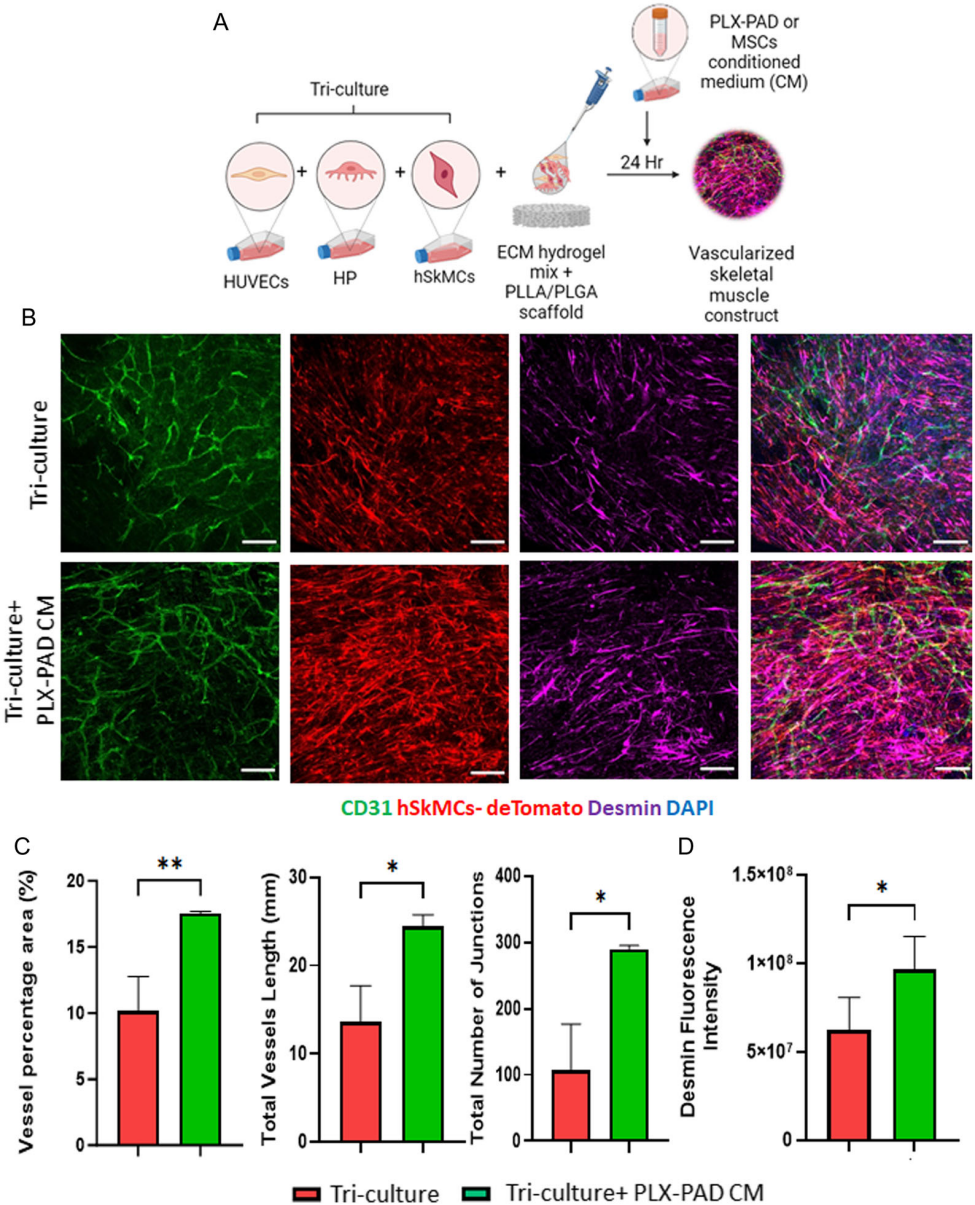
Hypoxic PLX‐PAD cell‐conditioned medium promotes vascular network formation and hSkMC differentiation in tri‐cultured system. A) Schematic presentation of the experimental procedure. hSkMCs expressing tdTomato were co‐seeded with endothelial cells, supporting cells, and hydrogel ECM mix on PLLA/PLGA scaffolds, with or without medium collected from PLX‐PAD cells. B) Representative confocal microscopy images of PLLA/PLGA scaffolds embedded with HUVECs, HP, and hSKMCs, with and without conditioned medium collected from PLX‐PAD cells cultured for 24 h under hypoxic conditions (1% of O_2_). On day 9 post‐seeding, samples were stained for CD31 (green), hSKMCs‐tdTomato (red), desmin (magenta) cell nuclei (DAPI). Scale bar = 200 μm. C) Vessel percentage area, total vessel length and total number of junctions on day 9 post‐seeding were determined using the AngioTool program. D) Desmin fluorescence intensity qualification. Data are presented as mean ± SD, *n* ≥ 3 per group. Significance of differences across groups was assessed by the *t*‐test. *p < 0.05, **p < 0.01.

Following failure of PLX‐PAD cells to support formation of well‐developed vasculature, it was hypothesized that their angiogenic effect could be activated under hypoxic conditions that mimic ischemic inflammatory conditions and stimulate secretion of proangiogenic factors (Figure S3, Supporting Information). It is known that hypoxic conditions alone induce angiogenesis and vascularization.^[^ ^3^, ^4^ ^]^ To isolate and study in a controlled manner the effect of hypoxia on the vascularization‐supporting capacity of PLX‐PAD cells secretome, the medium of cells grown under hypoxic conditions was collected and placed over a 3D system with HUVECs and HP (**Figure** **4**A). Scaffolds seeded with HUVECs and HP and later exposed to PLX‐PAD CM showed significantly more mature formation of the vascular network in comparison to those treated with MSCs CM (Figure 4B,C). Further, the HPs organized around the vasculature (Figure S4, Supporting Information), suggesting their role in supporting vascular network stabilization and maturation. Cryosection staining indicates a capillary lumen (Figure 4D). The CM of PLX‐PAD cells collected after 24 h of cultivation under hypoxic conditions, contained significantly higher levels of pro‐angiogenic factors such as angiogenin, angiopoietin‐2, EGF, bFGF, osteopontin, and leptin as compared to MSC CM (Figure 4E and S5, Supporting Information). In contrast, MSC CM contained significantly higher levels of other growth factors such as HB‐EGF, HGF, and PIGF (Figure S5, Supporting Information). The expression levels of PDGF‐BB and VEGF‐A were similar in both groups (Figure 4E). Next, we examine the effect of, the CM collected from PLX‐PAD cultured under hypoxia on a tri‐culture system comprised of hSkMC, HUVEC, and HP to form vascular skeletal muscle tissue (**Figure** **5**A). The collected CM significantly promoted vessel network formation within a tri‐culture system (Figure 5B,C). Also, desmin expression levels were significantly higher in samples treated with PLX‐PAD CM as compared to those without (Figure 5B,D). Previous studies show that the myogenesis process under hypoxic conditions (1% O_2_) is less substantial, with poorly organized fibers that, have smaller cross‐sections than their counterparts that develop in 21% O_2_.^[^ ^5^ ^]^ Therefore the addition of the hypoxic stimulated CM employed an alternative solution that allowed us to isolate PLX‐PAD cells’ influence.

Figure 4PLX‐PAD‐conditioned medium promotes vascular network formation. A) Schematic presentation of the experimental procedure. HUVECs were co‐seeded with HP and ECM hydrogel on PLLA/PLGA scaffolds. After 24 h the medium was changed to conditioned medium (CM) collected from PLX‐PAD cells or MSCs as a control cultured for 24 h under hypoxic conditions (1% O_2_). B) Representative confocal microscopy images of PLLA/PLGA scaffolds embedded with HUVECs and HP, stained for CD31 (green) and nuclei (blue) on day 7 post‐seeding. Scale bar = 200 μm. C) Average vessel length, total number of junctions and vessel percentage area were determined on day 7 post‐seeding, using the AngioTool program. Data are presented as mean ± SD, *n* ≥ 4. Statistical significance of the differences between groups was assessed by two‐way ANOVA. *p < 0.05, **p < 0.01. D) Representative confocal microscopy images of cryosectioned PLLA/PLGA scaffolds embedded with HUVECs and HP, and stained for CD31 (green) and nuclei (blue) on day 7 post‐seeding. Scale bar = 20 μm. E) Proangiogenic protein content of CMs was assayed by ELISA. Significance of differences between conditions was tested by *t*‐test. Data are represented as mean ± SD, *n* ≥ 4 per group. *p < 0.05, **p < 0.01, ***p < 0.001, ****p < 0.0001.

**Figure:**
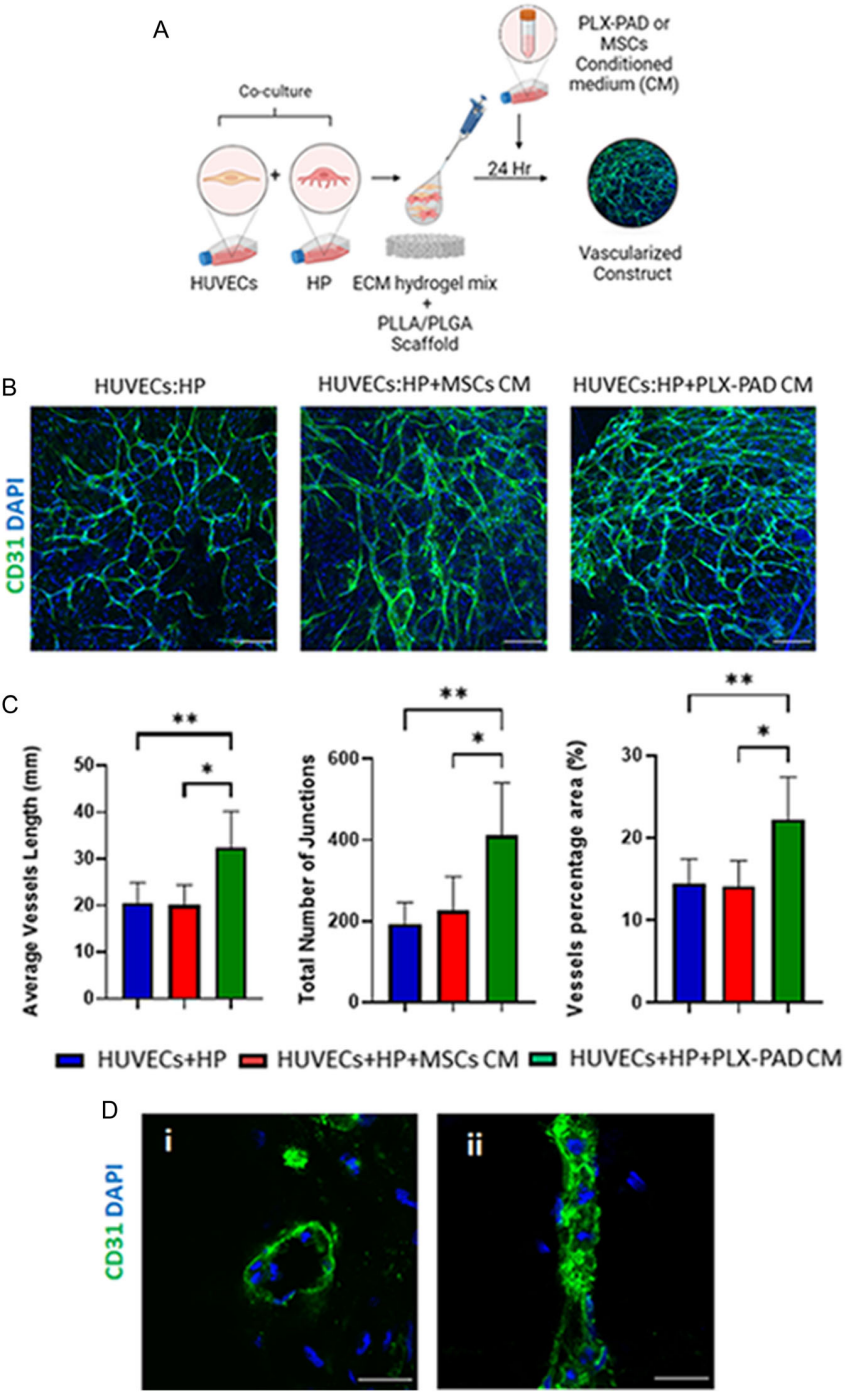

**Figure:**
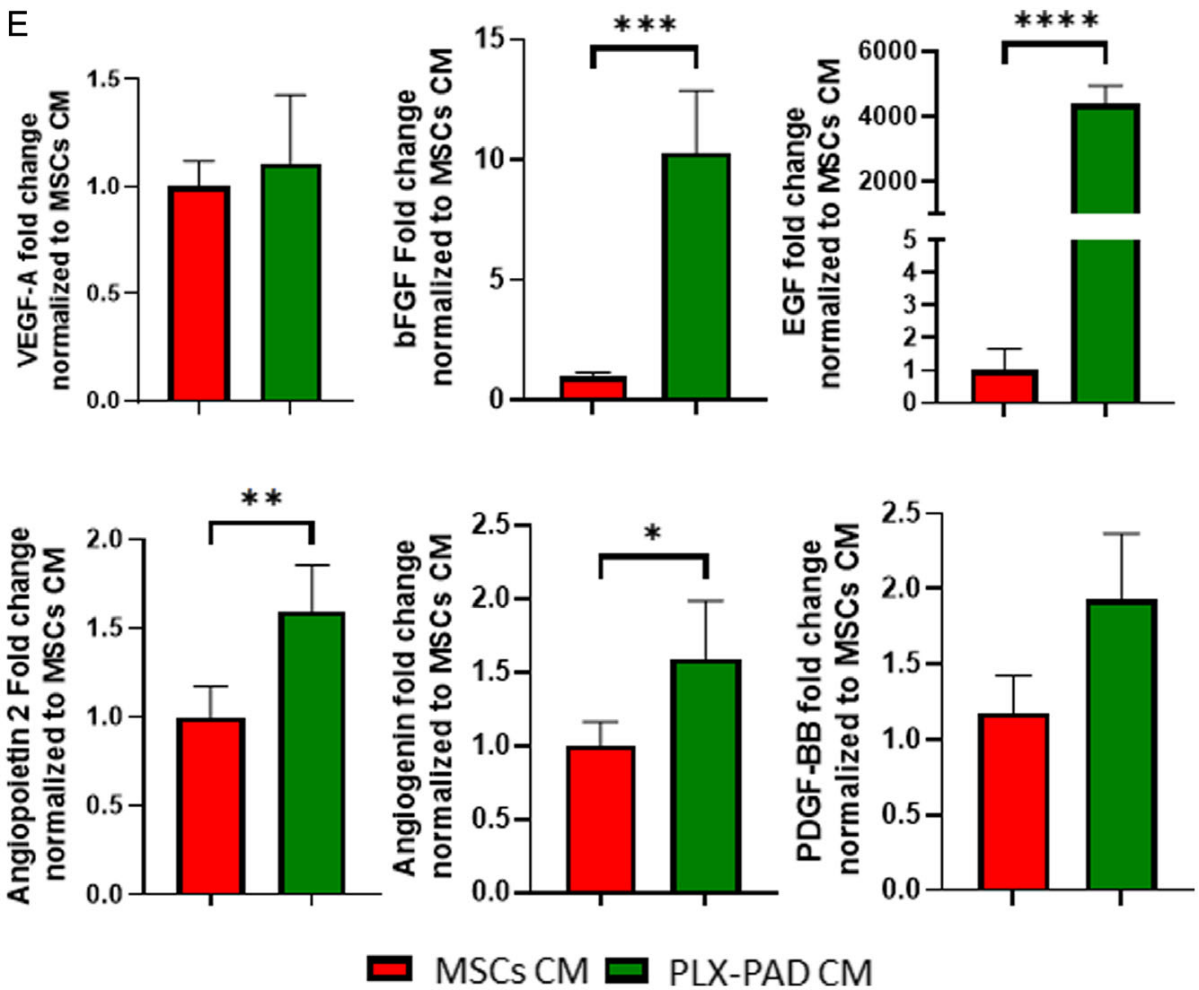

### 2.5 In Vivo Integration and Functionality of Implanted Vascularized Skeletal Muscle Graft

**Figure 6:**
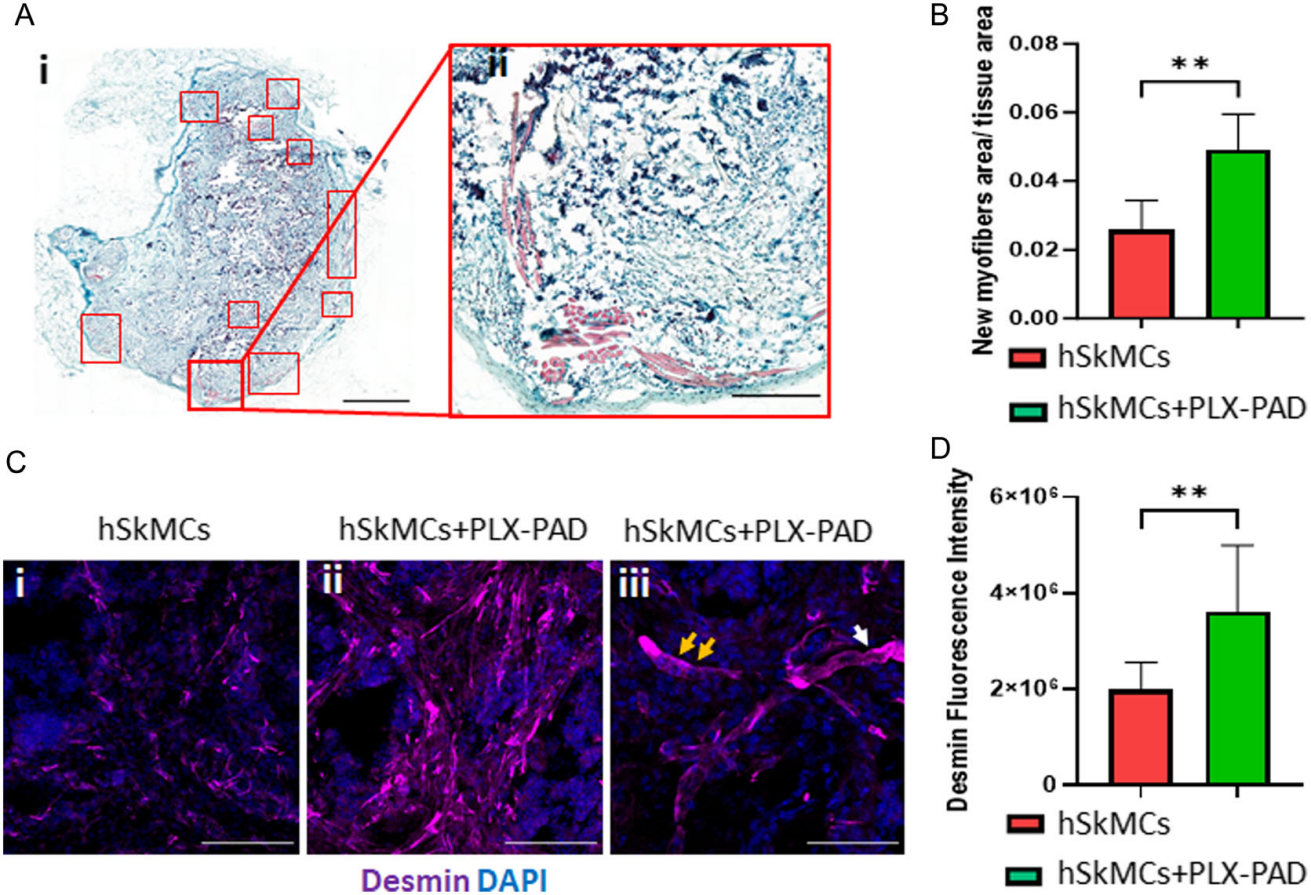
De‐novo muscle formation and hSkMC differentiation in vivo. A) Representative images of Masson's trichrome‐stained scaffolds embedded with hSkMCs and PLX‐PAD extracted 21 days after transplantation into 8‐week‐old nude mice. Red squares mark newly formed muscle bundles. Scale bar = 1000 μm i). Representative magnified images with newly formed muscle bundles stained light purple. Scale bar = 200 μm ii). B) New myofiber area inside the implanted scaffolds was determined using the Qupath program, on day 21 post‐implantation. Data are presented as mean ± SD, *n* ≥ 5 per group. Significance of differences between groups was estimated by the *t*‐test. **p < 0.01. C) i,ii) Representative images of cryosectioned PLLA/PLGA scaffolds immunostained for desmin and cell nuclei (DAPI). Scale bar = 200 μm. iii) Representative magnified images of striations in the muscle fibers, indicated by the white arrow, and multi‐nucleated muscle fiber, indicated by the yellow arrows. Scale bar = 50 μm. D) Desmin fluorescence intensity. Data are presented as mean ± SD, *n* ≥ 5 per group. Significance of differences between groups was estimated by the *t*‐test. **p < 0.01.

**Figure 7:**
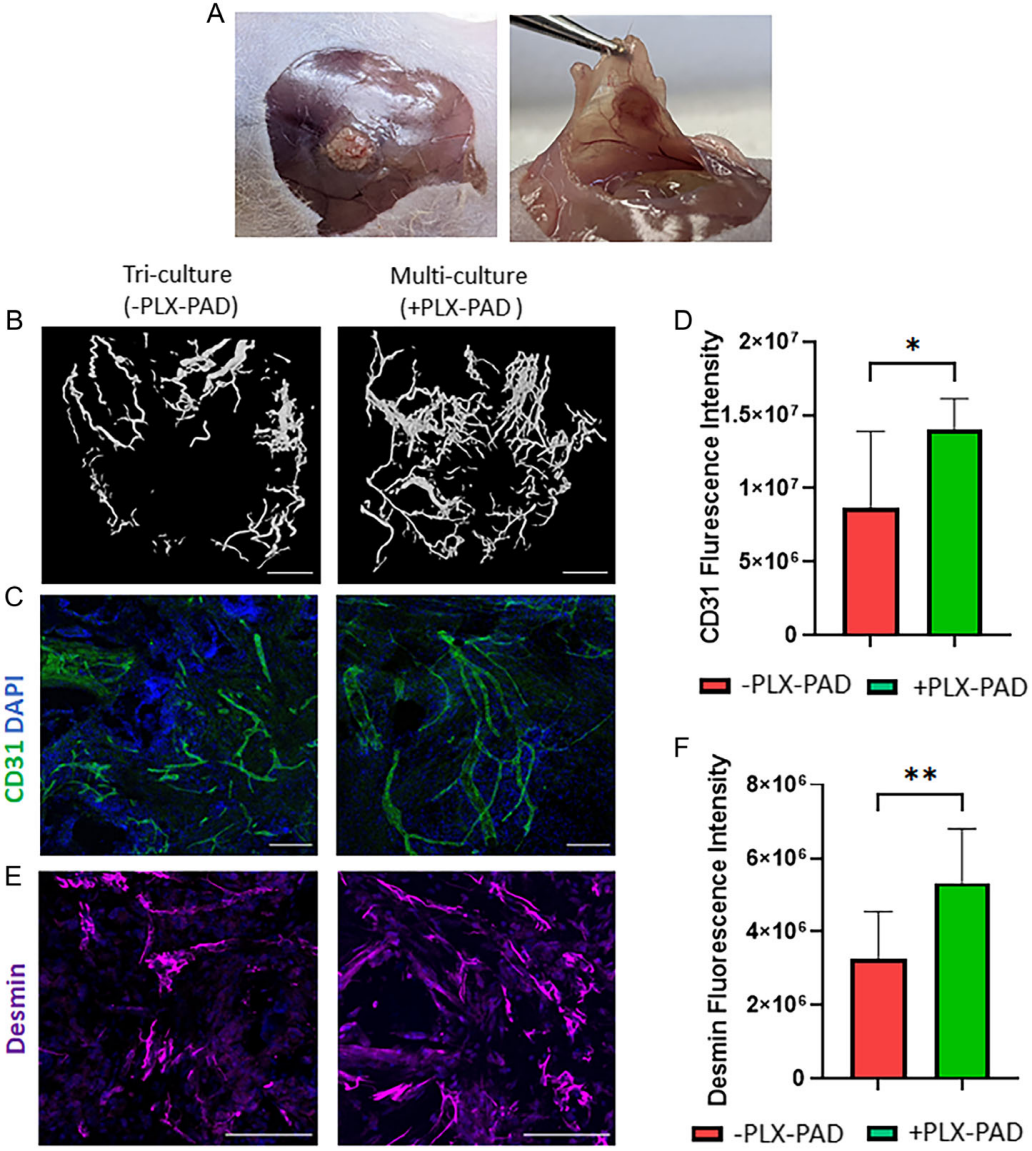
PLX‐PAD promote penetration of host's blood vessels into the vascularized skeletal muscle constructs and muscle differentiation. Ex‐vivo vascularized skeletal muscle tissue constructs 21 days after transplantation into 8‐week‐old nude mice. A) Penetration of host blood vessels into the graft. B) Representative μCT images of the penetrated blood vessels. Scale bar = 1000 μm. C,D) Representative images and fluorescence intensity quantification of cryosectioned constructs immunostained for CD31. Scale bar = 200 μm. E) Representative images of cryosectioned PLLA/PLGA scaffolds immunostained for desmin and cell nuclei (DAPI). Scale bar = 200 μm F) Desmin fluorescence intensity qualification. Data are presented as mean ± SD, *n* ≥ 5 per group. Significance of differences between groups was estimated by the *t*‐test. *p < 0.05, **p < 0.01.

After confirming that PLX‐PAD cells directly stimulate myogenic differentiation and that the CM of hypoxic PLX‐PAD promotes the maturation of vascular network in‐vitro, the in vivo effects of these cells were studied. Engineered muscle tissue composed of PLLA/PLGA scaffolds seeded with hSkMCs and PLX‐PAD cells, and transplanted into a rectus abdominal muscle defect site, had more newly formed muscle bundles on day 21 post‐implantation compared to control (hSkMCs only) (**Figure** **6**A,B). Additionally, the cryosectioned scaffolds showed higher desmin expression levels in comparison to the control group (Figure 6C,D), additionally, myoblast fusion was observed (Figure 6C). Next, engineered vascularized skeletal muscle tissues with and without PLX‐PAD cells were fabricated and implanted 10 days post‐seeding in a rectus abdominal muscle defect site. Functional host vessels were identified within the transplanted vascularized muscle tissue, with a larger amount in the scaffold with PLX‐PAD cells, especially in its center (**Figure** **7**A,B and S6, Supporting Information). CD31 and desmin expression levels were significantly higher in the implanted multi‐culture tissue bearing PLX‐PAD cells in comparison to the tri‐cultured scaffolds without PLX‐PAD cells (Figure 7C–F). There is evidence of survival of the human cells within the construct, 21 days after implantation, as seen in the scaffolds stained positively for the human‐specific nuclear marker (HuNu) (Figure S7, Supporting Information).

## 3 Discussion

This work investigated the effect of placenta‐derived MSC‐like cells on the vascularization and muscle maturation of engineered skeletal muscle tissue. Reducing the in vitro maturation time of the engineered tissue implant is critical to benefit the patients and reduce the cost of the fabrication process. Many works have demonstrated that hSkMC differentiation requires more time than vascularization.^[^ ^4^, ^20^, ^23^, ^25^, ^29^, ^30^, ^31^ ^]^ Hence, fast and robust muscle differentiation protocols are a significant advantage when attempting to fabricate vascularized skeletal muscle tissue for implantation. A co‐culture of myoblasts with MSCs was shown to increase myoblast proliferation.^[^ ^6^, ^31^ ^]^ Additionally, MSCs are known to support microcapillaries self‐assembly.^[^ ^21^, ^32^, ^33^ ^]^ Hence, this work focused on the potential utility of PLX‐PAD cells in enhancing muscle tissue differentiation and vascularization in vascularized skeletal muscle tissue engineering. We developed a co‐culture construct of hSkMCs and PLX‐PAD cells or MSCs as a control with fibrin‐based hydrogel on interconnected microporous 3D PLLA/PLGA biodegradable polymer scaffolds in vitro. In addition, we established constructs to study the development of engineered blood vessel networks by co‐culturing endothelial cells (HUVECs) with HP, MSCs, or PLX‐PAD cells using fibrin‐based hydrogel on PLLA/PLGA scaffolds. PLX‐PAD cells significantly enhanced the differentiation and maturation of the engineered skeletal muscle. However, PLX‐PAD cells did not directly promote vascular network formation. PLX‐PAD cells have minimal potential to differentiate into mural cells,^[^ ^2^ ^]^ which may explain their limited contribution to vascular network formation within co‐culture systems. These findings were further confirmed in a multi‐culture condition. The combination of HUVECs and HP which formed the microcapillaries and hSkMCs which formed the skeletal muscle fibers together with PLX‐PAD cells resulted in enhanced skeletal muscle cells differentiation without an additional effect on vascularization.

Under normoxic conditions, PLX‐PAD cells had a negligible effect on the formation of microcapillaries. In contrast, the CM of PLX‐PAD cells exposed to hypoxic conditions, which mimic states of ischemic inflammation, such as injury or disease,^[^ ^14^, ^34^ ^]^ significantly stimulated the formation of mature vascular networks. These findings align with previous reports of MSC secretome‐driven stimulation of angiogenesis under hypoxic conditions.^[^ ^3^, ^35^ ^]^ The effects were likely mediated by proangiogenic cytokines secreted into the CM in response to the hypoxic stress. Indeed, the expression of proangiogenic cytokines such as bFGF, EGF, angiopoietin 2, angiogenin and PDGF‐BB was significantly higher in PLX‐PAD CM as compared to MSCs CM. In contrast, HB‐EGF, HGF and PIGF expression was significantly higher in the CM of hypoxic MSCs, suggesting that the two cell types stimulate angiogenesis via distinct pathways.^[^ ^36^, ^37^ ^]^ Interestingly, the expression levels of bFGF and EGF were more dramatic, ≈10‐fold and more than three orders of magnitude respectively. It is known in the literature that bFGF and EGF are considered two critical factors in promoting angiogenesis.^[^ ^38^ ^]^ These factors can crosstalk with each other and with other proangiogenic pathways such as the VEGF and angiopoietin‐2 pathways.^[^ ^39^, ^40^, ^41^ ^]^ Besides being proangiogenic biomolecules bFGF and EGF can also stimulate cell proliferation, differentiation, and wound healing.^[^ ^42^ ^]^ These findings could offer a clue as to the significant effect of PLX‐PAD on angiogenesis compared to MSCs. This approach demonstrates the feasibility of achieving a mature vascular network by the addition of PLX‐PAD CM, thereby eliminating the necessity for direct cell culturing as required by conventional protocols.

To further improve vessel network fabrication in vitro, the hydrogel embedded with the cells was modified. We examined the influence of different hydrogels on our system, and the formulated ECM mix led to a significant positive effect on vascularization. Hence, we continued with this ECM hydrogel mix in our experimental system. containing fibrin, collagen, fibronectin and factor XIII that mimics the ECM composition in the healing site of injury. The ECM hydrogel mix components supply the physical and mechanical properties required to promote cellular growth, organization, stabilization, maturation, and function^[^ ^43^, ^44^, ^45^, ^46^, ^47^, ^48^, ^49^, ^50^ ^]^ which enhance vascular network formation within the compromised area. The unique ECM of healing tissue is a dynamic fibrin‐based matrix,^[^ ^51^ ^]^ in which fibrin is primarily cross‐linked with thrombin and covalently crosslinked with factor XIII. Its branched structure promotes growth factor and cell attachment and both facilitate permanent ECM deposition. The addition of factor XIII prolongs this mesh‐like effect and provides ideal conditions for tissue recovery.^[^ ^46^ ^]^ After the healing process is complete, the native ECM in various tissues is predominantly composed of collagen,^[^ ^47^, ^48^, ^49^ ^]^ which comprises the main component of the connective tissues within the skeletal muscle.^[^ ^52^ ^]^

When implanted into a rectus abdominal muscle defect in nude mice, PLX‐PAD cells enhanced invasion and perfusion of engineered multi‐culture scaffolds by host vessels. The induced injury likely triggered inflammatory stress and hypoxic conditions^[^ ^14^, ^34^ ^]^ in the defect area, which invoked a PLX‐PAD cell paracrine response similar to that observed under hypoxic conditions in vitro. These secretions stimulate the host ECs to sprout and self‐assemble into microcapillaries, penetrate the implanted tissue, and replace the engineered vasculature over time. A better differentiation in the presence of PLX‐PAD was observed. In previous studies in our lab, we sawed that there is a positive correlation between muscle differentiation and its functionality,^[^ ^53^, ^54^ ^]^ indicating the potential for our system to offer faster differentiation into functional muscle tissue. Moreover, the established immunosuppressive effect of PLX‐PAD cells^[^ ^5^, ^55^ ^]^ may have promoted integration of the implanted tissue with the host as well as new muscle formation.

## 4 Conclusions

This work presented a means of promoting engineered skeletal muscle maturation and vascularization by placenta‐derived MSC‐like cells. The placenta cells imparted a dual impact on myogenesis and vascularization, via both direct interaction and via secreted angiogenic factors. The study findings hold the potential to significantly enhance the production of engineered vascularized skeletal muscle tissue grafts with improved vascular networks, which can be more effectively transplanted and integrated in vivo. This advancement promises substantial improvements in regenerative medicine applications, facilitating more effective transplantation and in vivo integration of engineered muscle tissue.

## 5 Experimental Section

5.1

5.1.1

##### Cell Culture

Cells were thawed and seeded in 75 or 150 cm^2^ cell culture flasks (Techno Plastic Products AG, Switzerland). All cells were cultured in 5% CO_2_, humidified incubators at 37 °C. The medium was replaced every 2–3 days. All cells were used between passages 5–8 unless specified otherwise. Human umbilical vein endothelial cells (HUVECs) expressing green fluorescent protein (HUVEC‐GFP; Angio‐Proteomie, USA) were cultivated in endothelial cell medium (ScienceCell, USA), supplemented with 5% fetal bovine serum (FBS; ScienceCell), endothelial cell growth supplement (ScienceCell) and 1% penicillin‐streptomycin solution (PenStrep; ScienceCell). Pericytes isolated from human neonatal foreskin tissue (HP‐FS) were kindly provided by the Pritinder Kaur lab (Curtin University, Australia) and cultured in endothelial cell medium supplemented as previously described. Adipose‐derived MSCs (Lonza) were cultured in low‐glucose Dulbecco's modified Eagle medium (DMEM), supplemented with 10% FBS, 1% Glutamax, 1% Pen/Strep, and 10 mm HEPES. PLX‐PAD (Pluri Biotech Ltd., Haifa, Israel) were derived from single donor placenta from maternal origin as was described and characterized in previous studies^[^ ^4^, ^5^, ^56^ ^]^ extracted from healthy women undergoing elective cesarean sections. PLX‐PAD cells were cultured in low‐glucose DMEM supplemented with 10% FBS, 1% glutamine, and 0.1% gentamicin. Cells were harvested for experiments during passages 1–2. Human myoblasts (ScienCell), known as human skeletal muscle cells (hSkMC), were cultured in hSkMC medium (ScienCell) supplemented with 10% FBS, 1% Pen/Strep solution, and skeletal muscle growth factors, all provided in the medium kit. For differentiation, the cells were cultured in DMEM with 5% horse serum (Biological Industries) and 1% Pen/Strep solution. The hSkMC cells were harvested for experiments at ≈70% confluence.

##### Lentivirus Packaging and hSkMC Transduction with TdTomato Fluorescent Protein

Lentiviral particles were produced in T293 cells that were transduced with the pGenlenti td‐Tomato plasmid using the Lenti‐X universal packaging system (Takara Bio, USA), according to the manufacturer's instructions. Myoblasts were seeded at a concentration of 5 × 10^4^ cells per well in a 6‐well plate. The following day, they were incubated with the viral particles in the presence of 6 μg mL^−1^ polybrene (Sigma‐Aldrich), for 72 h, with medium replaced every 24 h to allow for expansion. Stable‐expressing cells were positively selected using puromycin‐supplemented plates (1.5 μg mL^−1^).

##### Scaffold Fabrication

Microporous scaffolds were prepared from 50% poly(L‐lactide) (PLLA) (Polysciences, Warrington) and 50% poly(lactic*‐co*‐glycolic acid) (PLGA) (Boehringer Ingelheim) using a salt‐leaching technique to acquire 93% porosity and pores between 212 and 600 μm.^[^ ^22^, ^28^ ^]^ PLLA and PLGA were dissolved 1:1 in chloroform to produce a 5% w/v polymer solution. Thereafter, 705 μl of the polymer mixture was transferred to Teflon cylinder molds (18 mm internal diameter) filled with 1.174 g sodium chloride porogenic particles, and were allowed to evaporate overnight. The resulting sponges were immersed for 8 h in distilled water (changed every hour) to leach out the salt and create an interconnected porous structure. The scaffolds were then dried and frozen overnight at −80 °C. Finally, the scaffolds were lyophilized overnight and kept dry under a vacuum until use. One day before seeding, 6 mm diameter and 1 mm thick round pieces were cut out with a biopsy punch (IntegraMiltex) and sterilized in 70% ethanol overnight. Before seeding, the scaffolds were washed twice in phosphate‐buffered saline (PBS) (Sigma) and dried using a vacuum.

##### Engineered 3D Tissue Construction: Monoculture

hSkMCs were cultured to 70% confluency, harvested and then re‐suspended in 10 μL of a 1:1 mixture of 15 mg mL^−1^ fibrinogen and 10 U/ml thrombin (Evicel). Cells (0.5 × 10^6^) were then seeded on 6 mm‐diameter PLLA/PLGA scaffolds, and incubated in 12‐well non‐tissue culture plates for 30 min at 37 °C, 5% CO_2_. After gelation, 1 mL growth medium was added to each well. The medium was replaced with 1 mL differentiation medium 48 h after the seeding, which was replaced every other day.

##### Engineered 3D Tissue Construction: Co‐culture: Skeletal Muscle Construct

hSkMCs were co‐cultured with adipose‐derived MSCs or with PLX‐PAD cells in a 1:2 ratio. After harvest, the co‐culture (0.75 × 10^6^ cells) was resuspended in 10 μL of a 1:1 mixture of 15 mg mL^−1^ fibrinogen and a 10 U mL^−1^ thrombin, seeded onto 6 mm diameter PLLA/PLGA scaffolds, and incubated for 30 min, at 37 °C. After gelation, a hSkMC growth medium with MSCs medium in a ratio of 1:1 was added to each well. The medium was replaced 48 h after seeding, with 1 mL muscle differentiation medium with MSCs medium in a ratio of 1:1, and replaced thereafter every other day.

##### Engineered 3D Tissue Construction: Co‐culture: Vascularization Construct

HUVECs and pericytes or HUVECs and MSCs (adipose‐derived or PLX‐PAD) were co‐cultured at a 1:4 (0.1 × 10^6^ HUVECs and 0.4 × 10^6^ pericytes) or 1:3 (0.1 × 10^6^ HUVECs and 0.3 × 10^6^ MSCs) ratio, respectively on 6‐mm PLLA/PLGA scaffolds. The co‐culture was suspended in 10 μl of a 1:1 mixture of fibrin which contained 15 mg mL^−1^ fibrinogen and 10 U mL^−1^ thrombin or 2 mg mL^−1^ collagen type 1 (Corning) or hydrogel extracellular matrix (ECM) mix comprised of 5 mg mL^−1^ fibrinogen (Evicel), 5 U mL^−1^ thrombin (Evicel), 5 U mL^−1^ fibrogammin (FXIII, CSL Behring GmbH, Germany), 2 mg mL^−1^ collagen type 1 Corning) and 90 μg mL^−1^ fibronectin (Sigma) and seeded onto the scaffolds. After a 30 min incubation at 37 °C, endothelial cell medium with a MSCs medium in a ratio of 1:1 was added to each well. The medium was changed every other day.

##### Engineered 3D Tissue Construction: Tri‐culture/Multi‐Culture

HUVECs, HP‐FS, and hSkMCs were tri‐cultured at a 1:4:5 ratio (0.1 × 10^6^, 0.4 × 10^6^, 0.5 × 10^6^), respectively. For multi‐cultures including PLX‐PAD cells as well, the cell ratio was 1:4:5:2.5 (0.1 × 10^6^, 0.4 × 10^6^, 0.5 × 10^6^, 0.25 × 10^6^) for HUVECs, HP, hSkMCs and PLX‐PAD, respectively. Subsequently, cultures were harvested and resuspended in 10 μL ECM hydrogel mix, as described above. Scaffolds were crosslinked for 30 min at 37 °C, after which 1.5 mL of 1:1:1 EC medium‐skeletal muscle medium‐full DMEM were added to each well. The medium was changed every other day.

##### Collection of Conditioned Medium (CM)

PLX‐PAD cells and MSCs were seeded on six‐well culture plates (1 × 10^6^ cells/well) in full DMEM and incubated for 24 h at 37 °C, 21% O_2_% and 5% CO_2_ in a humidified incubator. After 24 h, each well was rinsed with 1 mL PBS, before being incubated with 1 mL endothelial basal medium (EBM‐2, Lonza) for an additional 24 h under hypoxic conditions at 37 °C, 1% O_2_% and 5% CO_2_ in a humidified incubator. Supernatant was collected and added to the HUVECs‐HP co‐culture system of or to the tri‐culture system described earlier, one day after they were seeded on the 3D PLLA/PLGA scaffolds.

##### Angiogenic ELISA Kits

The CM collected from PLX‐PAD cells and MSCs was subjected to semi‐quantitative human angiogenesis ELISA testing according to the manufacturer's instructions (RayBiotech).

##### Whole‐Mount Immunofluorescence Staining and Imaging

Whole scaffolds were washed with PBS and then fixed in 4% paraformaldehyde (PFA; Electron Microscopy Sciences) for 15 min, at room temperature. Scaffolds were then rinsed with PBS (5 min ×3), before being treated with 0.3% Triton X‐100 (Bio Lab Ltd.) for 10 min, to permeabilize the cell membrane. Thereafter, scaffolds were washed with PBS (5 min ×3) and incubated overnight at 4 °C with 5% bovine serum albumin (BSA) blocking solution (Merck Millipore). Samples were then incubated with primary antibodies diluted in the blocking solution: mouse anti CD31 (1:100; Dako, Agilent Technologies, USA), to mark endothelial cells, mouse anti‐α‐smooth muscle actin (α‐SMA) (1:50; Dako), to mark pericyte support cells, rabbit anti‐desmin (1:200 Abcam), to mark skeletal muscle, Mouse anti‐ HuNu (1:100 Abcam), to mark human nuclear membranes. The following day, the samples were washed (5 min ×3) with PBS and incubated for 3 h, at room temperature, with Alexa 488 goat anti‐mouse (1:400; Invitrogen) or Alexa 647‐ donkey anti‐rabbit (1:400; Invitrogen) antibodies diluted in PBS and mixed with 4',6‐diamidino‐2‐ phenylindole (DAPI) (1:1000; Sigma). Finally, the scaffolds were washed with PBS (5 min ×3) and stored at 4 °C until imaging. Constructs were imaged with a LSM700 confocal microscope (Zeiss, Germany), with 2.5×, 5×, 10×, 20× and 63× oil immersion lenses using the Zen software. Fiji software was used to create the maximum projection of the slices per image and to measure the fluorescence intensity.^[^ ^57^ ^]^ Vascularization analysis was performed using the AngioTool, a computational tool for quantitative analysis of vascular network parameters, such as average vessel length, the total number of junctions and vessel percentage area.^[^ ^58^ ^]^

##### Protein Extraction and Western Blot

Scaffolds were washed with PBS and homogenized in RIPA buffer (Sigma‐ Aldrich) supplemented with a protease inhibitors cocktail (100 mM PMSF, 2 mg mL^−1^ leupeptin, 50 mm Na‐orthavanadate, 50 mm aprotinin (Sigma‐Aldrich)). Total protein content was determined using the BCA protein assay kit (Thermo Scientific). Equal amounts of protein from each sample were resolved on 10% sodium dodecyl sulfate‐PAGE (SDS‐PAGE) gels (Invitrogen NuPage, Life Technologies)and blotted onto nitrocellulose membranes (Bio‐Rad, Hercules, CA). Membranes were blocked with blocking buffer (Bio‐Rad, Hercules, CA), and then immunoblotted (overnight at 4 °C) with primary antibody: 1:500 rabbit anti‐desmin (Abcam), or 1:1000 mouse anti‐GAPDH (Santa Cruz), diluted in blocking buffer. Bound antibodies were detected following incubation with horseradish peroxidase‐conjugated anti‐mouse/rabbit IgG (1:5000; GE Healthcare Life Sciences), followed by exposure to the enhanced chemiluminescence Western blotting system (ECL; Amersham Biosciences). Images were acquired with the LAS‐3000 imaging system (FujiFilm). Dense cytometry analysis was performed using Fiji software. Data are presented relative to GAPDH expression.

##### Transplantation of Engineered Tissue

All surgical procedures were conducted according to protocols approved by the Institutional Animal Care and Use Committee of the Technion (ethics number IL‐201‐12‐2022). PLLA/PLGA scaffolds cultured with hSkMCs, hSkMCs and PLX‐PAD cells, or hSkMCs, endothelial cells and pericytes with or without PLX‐PAD cells were implanted into male, 8‐week‐old nude mice (Harlan Laboratories) (*n* = 8 per group). All scaffolds were incubated for 10 days at 37 °C before implantation. Mice were anesthetized with 1–2% isoflurane at flow rate of 1 L min^−1^, after which, an incision was made in the ventral skin to expose the abdominal wall. Next, a 5‐mm‐diameter full‐thickness segment of the rectus abdominis muscle was removed and replaced with engineered skeletal muscle, which was secured with 8‐0 polypropylene sutures. Then, the skin was closed over the replaced abdominal tissue and sutured with 4‐0 silk sutures. Following the procedure, slow‐release buprenorphine (3.25 mg kg^−1^) is injected subcutaneously. All mice were closely monitored and sacrificed 3 weeks post‐surgery.

##### Immunohistochemistry and Immunofluorescence Analyses

The muscle with the implanted scaffold was retrieved 3 weeks post‐transplantation, dissected, rinsed in PBS, placed in 4% PFA for 25 min and rinsed 3 times with PBS for 5 min each. The scaffolds were then incubated overnight in a 30% w/v sucrose solution, embedded in optimal cutting temperature (OCT) compound (Tissue‐Tec, USA), and frozen for subsequent cryosectioning to 5, 20, and 150 μm thick sections. Briefly, for immunofluorescence staining, the sections were incubated in 0.5% Tween solution for 20 min, rinsed with PBS, and then blocked with 5% w/v BSA (Sigma‐Aldrich) for an additional 30 min. Subsequently, sections were incubated overnight at 4 °C with mouse‐anti CD31 antibody (1:100; Dako, Agilent Technologies, USA), to mark endothelial cells or rabbit‐anti desmin antibody (1:200 Abcam), to mark pericyte support cells. The following day, the samples were washed (5 min ×3) with PBS and incubated for 3 h, at room temperature, with Alexa 488 goat anti‐mouse (1:400; Invitrogen) or Alexa 647‐ donkey anti‐rabbit (1:400; Invitrogen) antibodies diluted in PBS and mixed with DAPI (1:1000; Sigma) before being mounted in Fluromount‐G (Southern Biotechnology) and examined under a confocal microscope. Additionally, the slides were rinsed with distilled water, stained with trichrome stain (Sigma‐Aldrich) for 2 min and then washed twice in 0.2% glacial acetic acid and then in DDW. Afterward, the slides were dehydrated by serial immersions in increasing concentrations of ethanol, and finally dipped in xylene and covered with Vectamount. The percentage of the muscle area formed de‐novo within the scaffold was determined using QuPath version 0.5.1.^[^ ^59^ ^]^ To this end, a pixel classifier was trained on several representative images with manually assigned annotations for de‐novo muscle, scaffold, hydrogel and background. The percentage of de‐novo muscle was calculated by dividing the area of the de‐novo muscle by the area of the scaffold and hydrogel.

##### In Vivo Vascularization and Micro‐Computed Tomography Scanning

At the end point of the experiment, i.e., 21 days post‐transplant, mice were anesthetized with ketamine and xylazine, after which, 10 mL warm heparinized saline followed by 10 mL Microfil (MV‐122, FlowTech Inc., Carver, MA) were transcardially perfused.^[^ ^16^ ^]^ Scaffolds were harvested and left to polymerize at 4 °C, overnight. Scaffolds were then extracted and immersed for 20 min in 4% PFA. High‐resolution micro‐CT scanning was performed with a Skyscan 1276 (Bruker, Kontich, Belgium), using the following parameters ‐ source voltage of 55 kV and source current of 72 μm filter aluminum 0.25 mm, rotation step angle 0.3° and frame averaging of 2 frames per angle with 950 ms exposure for each frame, and 360° scan for each sample. Images were acquired at a scaled pixel size of 11.75 μm. Scans were reconstructed using NRecon software (SkyScan, version 1.7.5.4), and DataViewer (SkyScan, version 1.5.6.5) was utilized to ensure proper alignment of the scaffold samples, which were then analyzed by CTAnn (SkyScan, version 1.19.8.0) using segmentation, and VOI for construction and analysis. 3D models were visualized using CTVox (SkyScan, version 3.3.0).

##### SEM Imaging

Scaffolds were dried by lyophilization and imaged with a Prisma scanning electron microscope (FEI) at 20 kV, spot size 4.5 nm. Samples were coated with Au/Pd under high‐vacuum conditions.

##### Statistical Analysis

The data are presented as mean ± SD. Group differences were assessed by a two‐tailed Student's *t*‐test. A two‐way analysis of variation (ANOVA) was performed to examine the influence of two independent categorical variables, followed by Bonferroni's multiple comparison test. *p* < 0.05 was considered significant. Statistical analyses were performed using GraphPad Software.

## Conflict of Interest

The authors declare no conflict of interest.

## Ethical Statement

The animal experiments were conducted according to protocols approved by the Institutional Animal Care and Use Committee of the Technion (ethics number IL‐201‐12‐2022).

## Supporting information

Supplementary Material

## Data Availability

The data that support the findings of this study are available from the corresponding author upon reasonable request.
