# Supplementary material for: Placenta‐Derived Mesenchymal Stromal‐Like Cells Promote 3D‐Engineered Muscle Tissue Differentiation and Vessel Network Maturation
Source: Small Sci. 2024 Aug 6;4(11):2400228. doi: 10.1002/smsc.202400228 (PMC11935177; doi:10.1002/smsc.202400228)
Supplement: Supplementary file 1 — Supplementary Material [file SMSC-4-2400228-s001.pdf]

Placenta-derived mesenchymal stromal-like cells promote 3D-engineered muscle tissue differentiation and vessel network maturation

Anna Tsukerman[1,2], Majd Machour[1], Margarita Shuhmaher[1], Eliana O. Fischer[1], Hagit Shoyhet[1,3], Orit Bar-Am[1], Gali Guterman Ram[1], Lior Debbi[1], Dina Safina[1], Shulamit Levenberg[1]

[1]Faculty of Biomedical Engineering, Technion-Israel Institute of Technology, Haifa, Israel.

[2]Interdisciplinary Program for Biotechnology Technion- Israel Institute of Technology, Haifa, Israel.

[3]Multidisciplinary Program for Nanoscience and Nanotechnology Technion- Israel Institute of Technology, Haifa, Israel

Supplementary

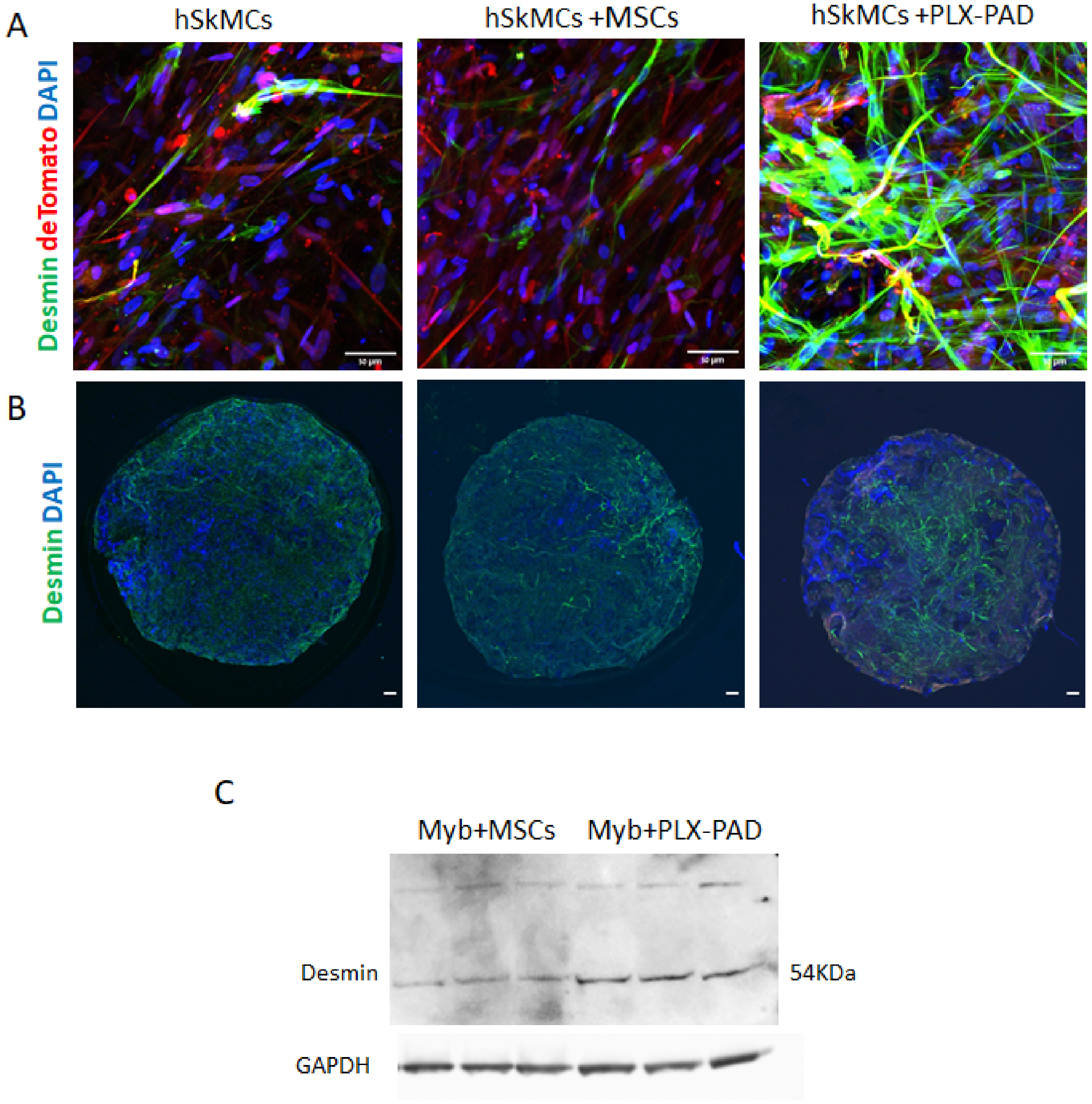

Figure S1. Desmin is highly expressed in a co-culture of hSkMCs and PLX-PAD cells. (A) *Representative confocal microscopy images of PLLA/PLGA scaffolds embedded with hSkMCs, co-culture of hSkMCs with PLX-PAD cells or hSkMCs with MSCs, stained for desmin (green) and nuclei (blue) 14 days post-seeding. Magnification, scale bar = 50 µm.* ***(B)*** *Whole-scaffold size image, scale bar = 200 µm.* ***(C)*** *Western blot gel demonstrating desmin expression in the scaffolds.*

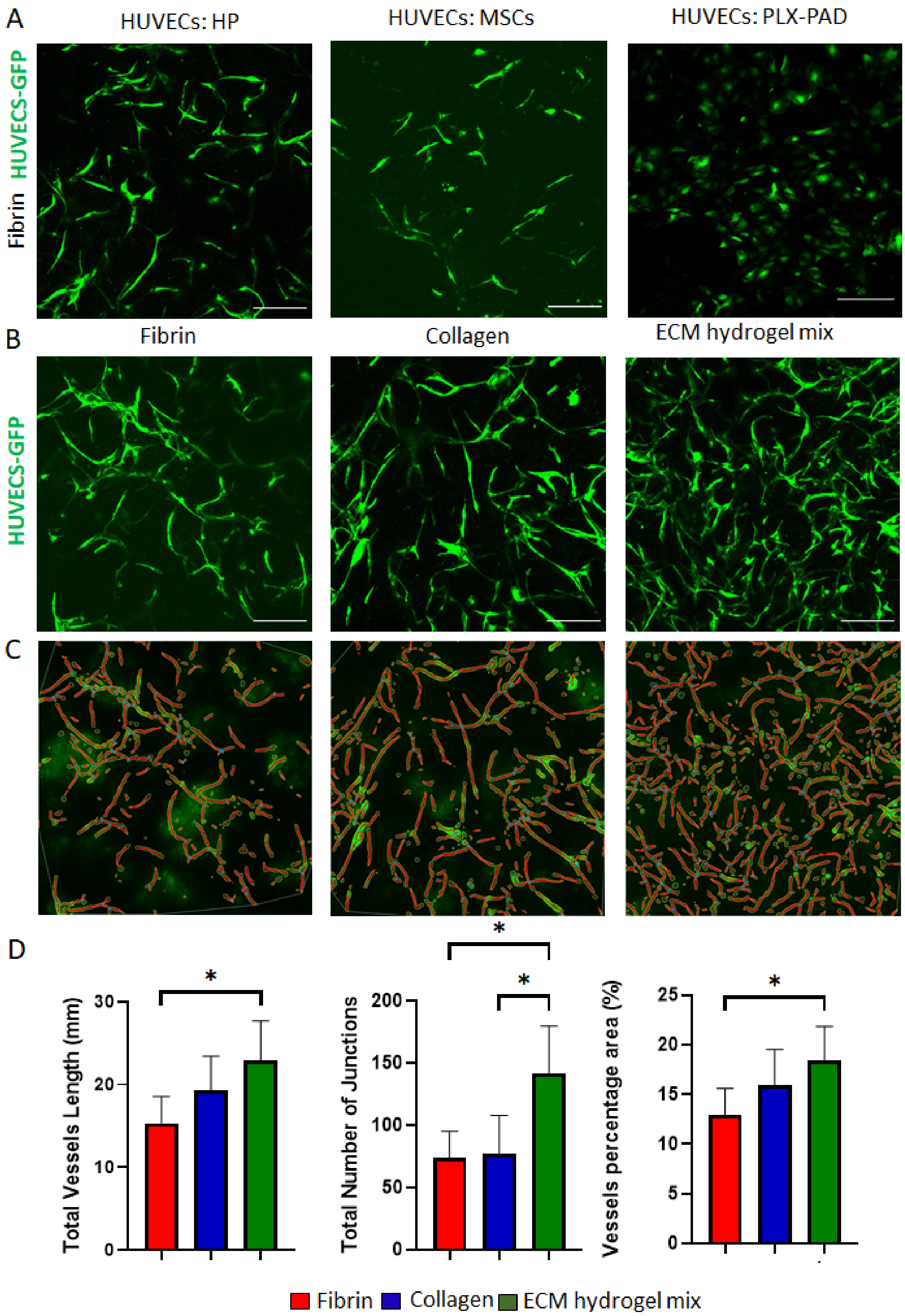

A
HUVECs: HP
HUVECs: MSCs
HUVECs: PLX-PAD
Fibrin HUVECS-GFP
B
Fibrin
Collagen
ECM hydrogel mix
HUVECS-GFP
C
D
Total Vessels Length (mm)
Total Number of Junctions
Vessels percentage area (%)
*
Fibrin
Collagen
ECM hydrogel mix

Figure S2. ECM hydrogel mix enhances vascular network development and maturation. (A) *Representative confocal images of fibrin hydrogel on PLLA/PLGA scaffolds embedded with HUVECs-GFP and different support cells, 7 days post-seeding. Scale bar = 200 µm* ***(B)*** *Representative confocal images of different hydrogels on PLLA/PLGA scaffolds embedded with HUVECs-GFP and HP cells, 7 days post-seeding. Scale bar = 200 µm.* ***(C)*** *Representative vascular network. Images were analyzed using the freeware AngioTool to quantify i) average vessel length, represented by the area circumscribed by the red lines following the network skeleton, ii) total number of junctions represented by blue dots, iii) vessel percentage area, represented by the area circumscribed by a yellow contour. The elements with no contour were ignored.* ***(D****) Average vessel length, total number of junctions and vessel percentage area, were estimated using the AngioTool program 7 days post-seeding. Data are presented as mean ± SD, n≥4 per group. Significance of differences across groups was estimated using Two-way ANOVA. *p<0.05.*

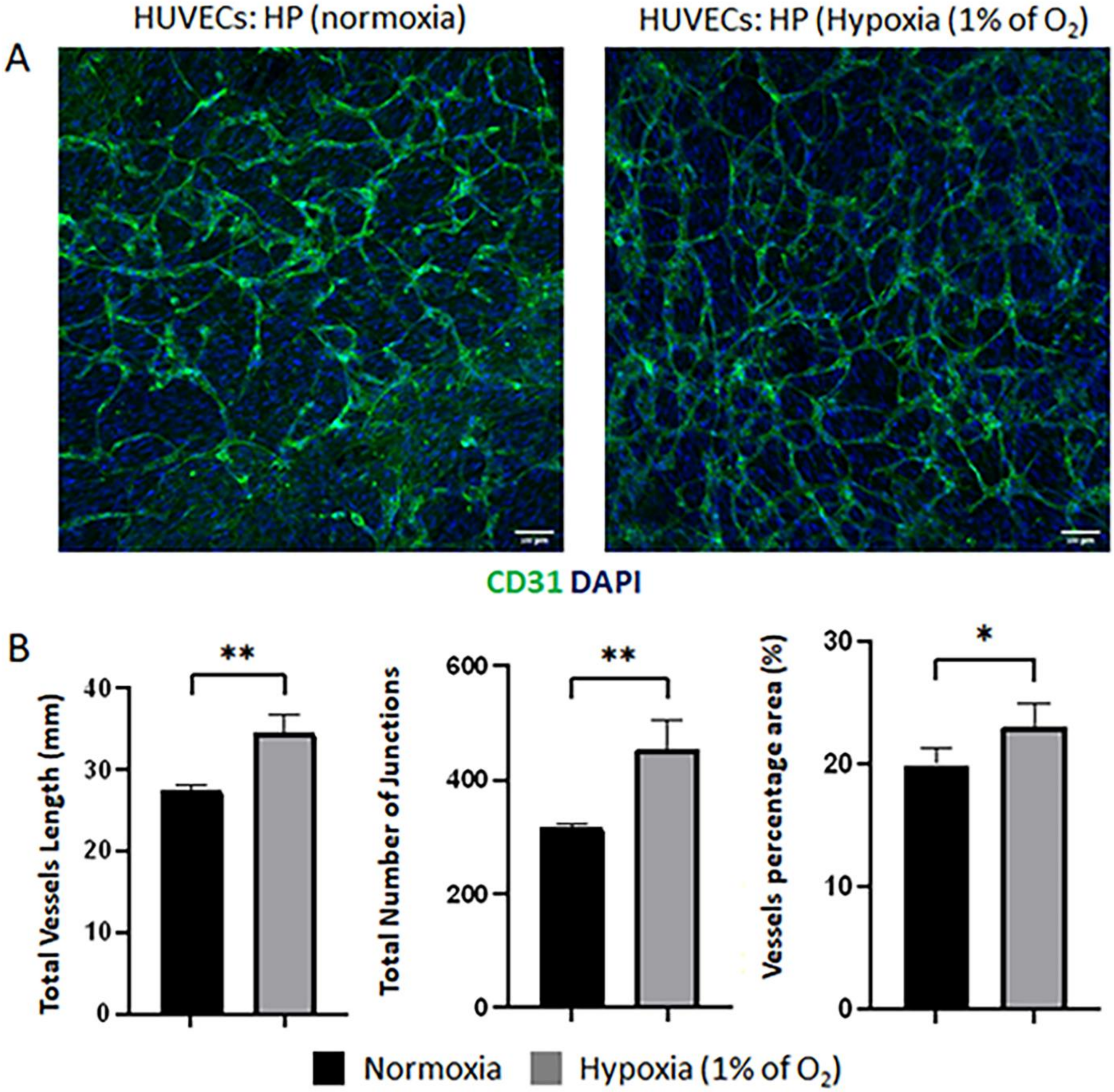

Figure S3. Hypoxic conditions stimulate vascularization. (A) *Representative confocal microscopy images of PLLA/PLGA scaffolds embedded with HUVECs and HP in normoxia or 24 h under hypoxic conditions (1% of O2). On day 7 post-seeding, samples were stained for CD31 (green) and cell nuclei (DAPI). Scale bar =100 µm.* ***(C)*** *Total vessel length, total number of junctions, and vessel percentage area, on day 7 post-seeding were determined using the AngioTool program. Data are presented as mean ± SD, n=4 per group. Significance of differences across groups was assessed by the t-test. *p<0.05, **p<0.01.*

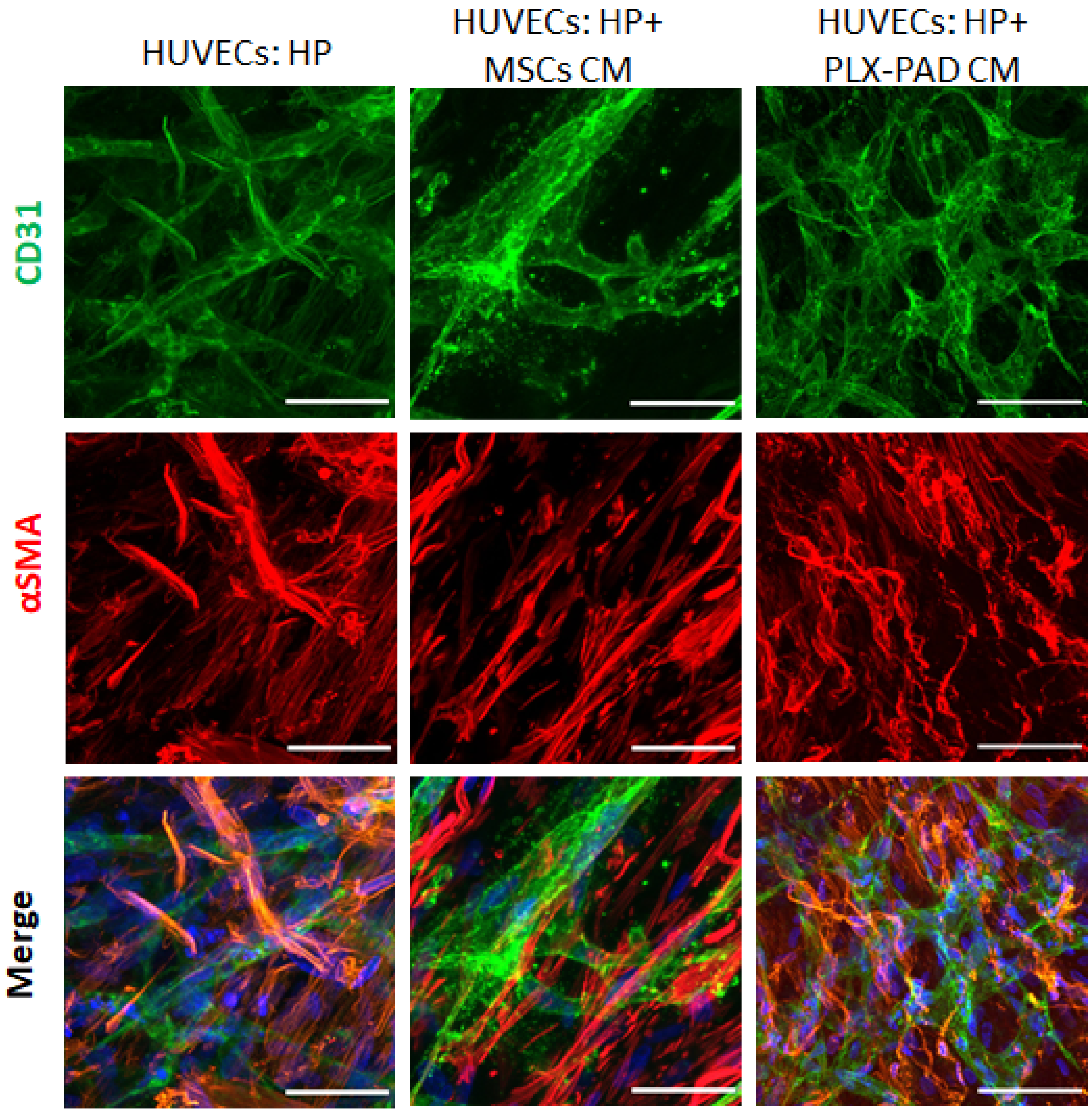

Figure S4. HP support vascular network formation. (A) *Representative confocal images of PLLA/PLGA scaffolds embedded with HUVECs and HP, stained for CD31 (green), alpha-smooth muscle actin (αSMA) (red), and nuclei (blue), 7 days post-seeding. Scale bar = 100 μm.*

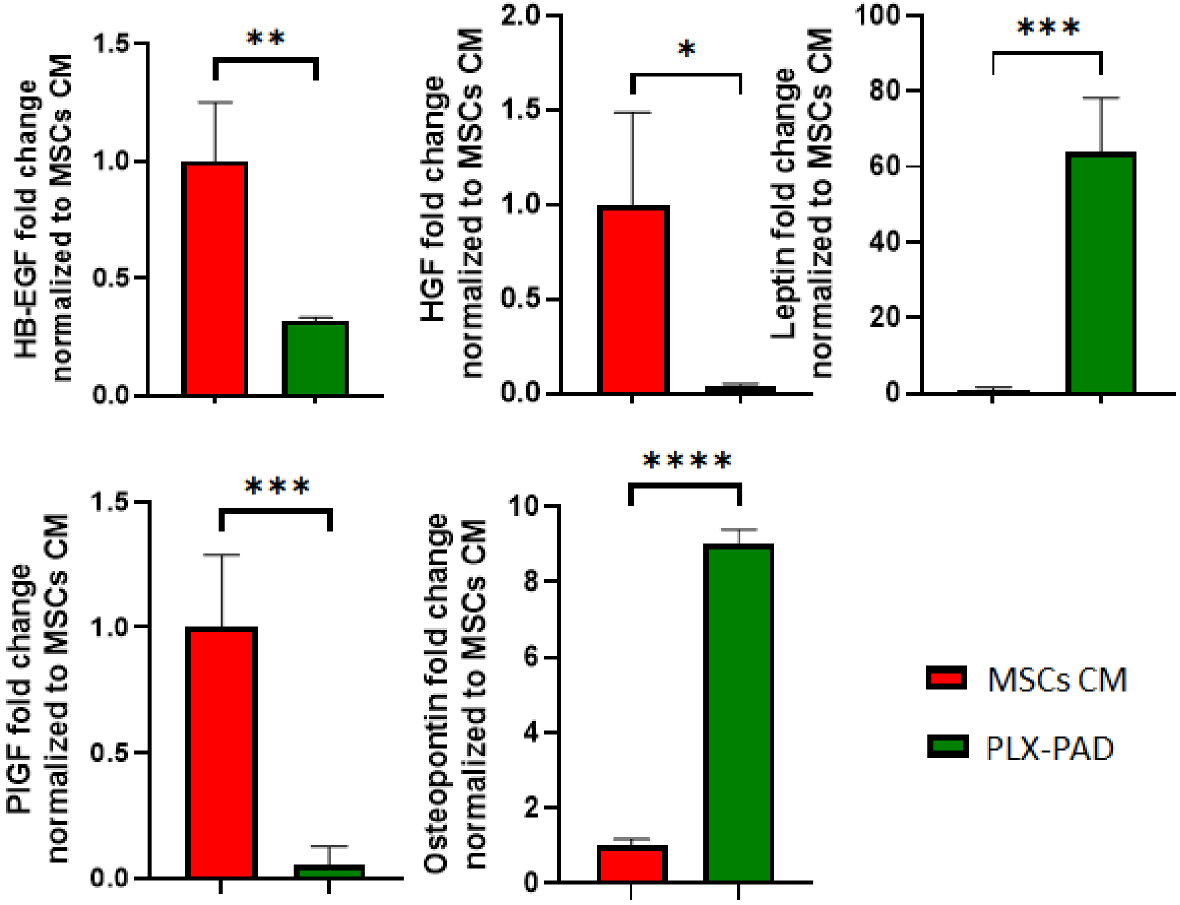

Figure S5. Proangiogenic proteins secreted by MSCs and PLX-PAD. *PLX-PAD cells and MSCs were cultured for 24 h under hypoxic conditions (1% of O2). Conditioned medium was collected, and proangiogenic protein contents were assayed by ELISA. Significance of differences between conditions was assessed by t-test. Data are presented as mean ± SD, n≥4 per group. *p<0.05, **p<0.01, ***p<0.001, ****p<0.0001.*

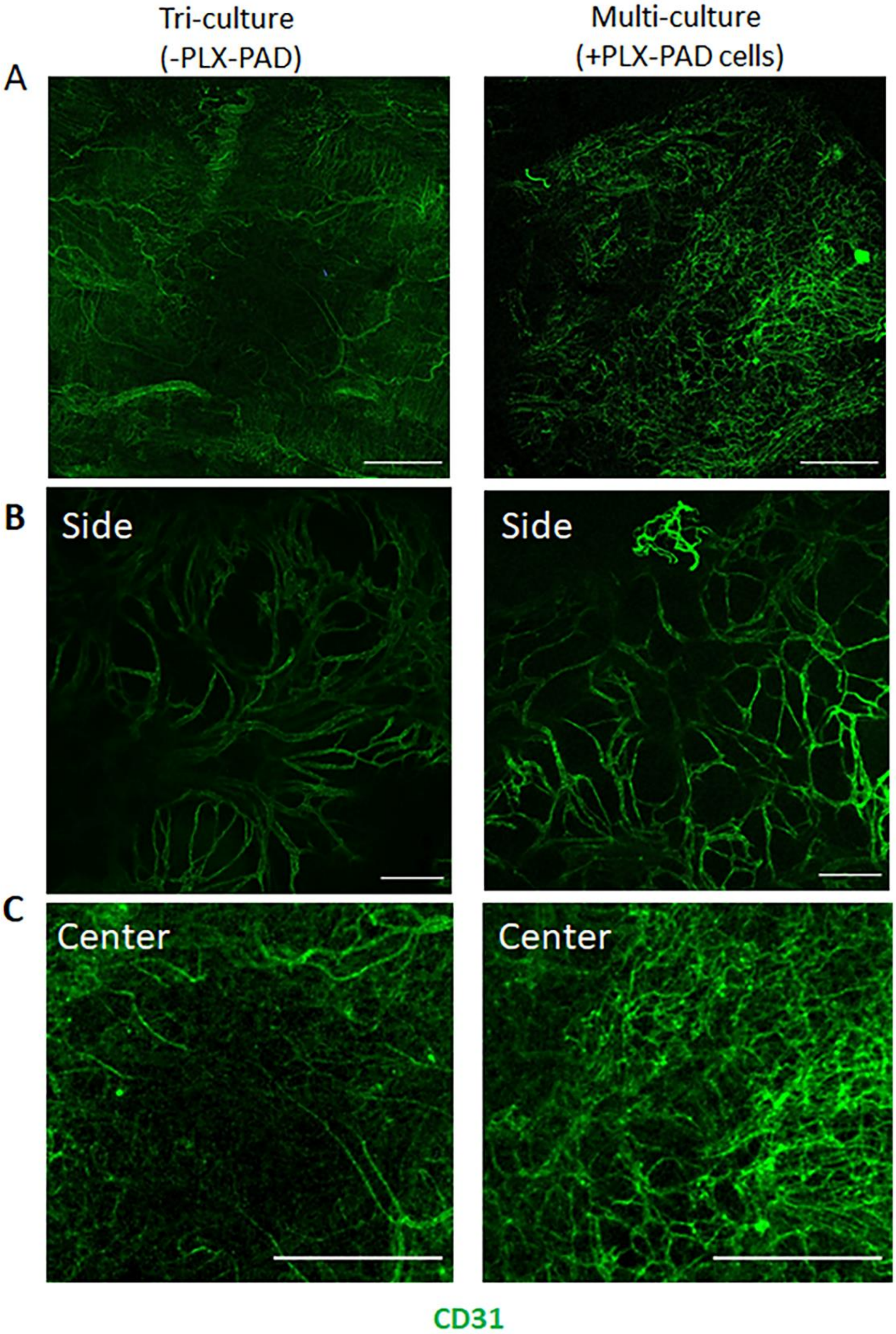

Figure S6. Perfusion of the transplanted vascularized skeletal muscle graft by host blood vessels. (A) *Representative images of perfusion of labeled CD31 antibodies injected to the host venous bloodstream into transplanted PLLA/PLGA constructs composed of a cell tri-culture without PLX-PAD (left), or a multi-culture with PLX-PAD (right). Scale bar = 1000 µm.* ***(B, C)*** *Representative magnified images of the vasculature inside the scaffold area, the side and the center of each scaffold. Scale bar = 200 µm and 1000 µm respectively.*

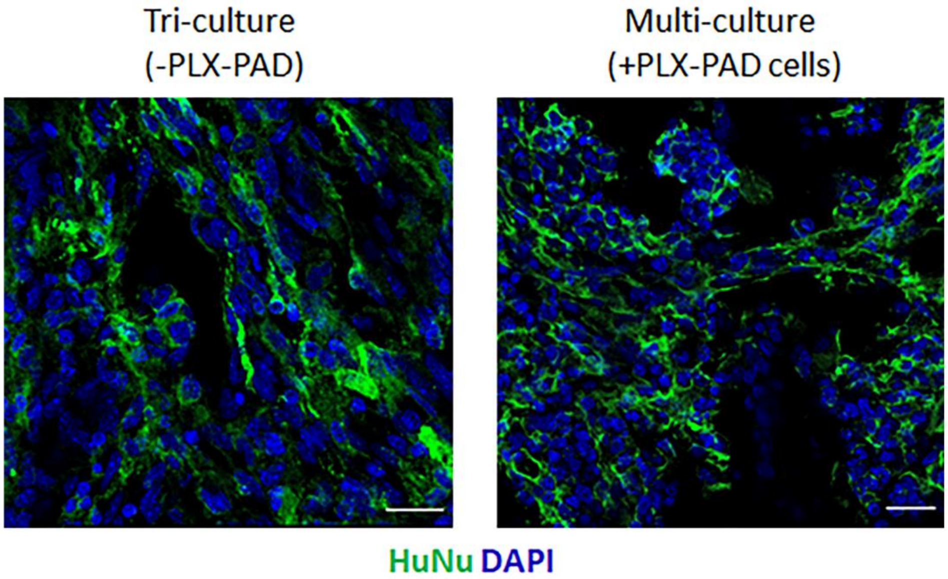

Figure S7. Human cells survived after transplantation. *Explanted vascularized skeletal muscle tissue constructs with and without PLX-PAD cells 21 days after transplantation into 8-week-old nude mice. Representative images of cryosectioned PLLA/PLGA scaffolds embedded with human cells immunostained for HuNu and cell nuclei (DAPI). Scale bar = 20 µm.*
